# A review of full-stack autonomous obstacle avoidance for assistive robots for the disabled

**DOI:** 10.3389/frobt.2026.1859583

**Published:** 2026-09-16

**Authors:** Yuan Zhang, Shuo Wang, Changlong Zhao, Wei Li, Zhenrong Shi, Meng Liu

**Affiliations:** College of Mechanical and Vehicle Engineering, Changchun University, Changchun, China

**Keywords:** environmental perception, multi-algorithm integration, obstacle recognition, path planning, scenario-based applications

## Abstract

With the intensification of population aging and the increasing awareness of protecting the rights of the disabled, significant progress has been made in assistive robots for visually impaired people, the mobility impairments and other groups. Research in this field focuses on four key technical dimensions: environmental perception, obstacle recognition and classification, path planning and obstacle avoidance algorithms, and scenario-based applications. This analysis particularly focuses on the ability of multi-sensor fusion to acquire spatial information in complex and dynamic environments, and reviews the technological evolution in target detection, semantic understanding, and passable area determination. The work further explores algorithms ranging from global path planning to local obstacle avoidance strategies, as well as reinforcement learning and multi-algorithm integration. Furthermore, this narrative review synthesizes the current research on assistive technologies, including guide robots for the visually impaired, intelligent mobility platforms for wheelchair users, and solutions adaptable to both indoor and outdoor environments. The insights derived from this review offer a reliable foundation to support the travel and daily activities of people with disabilities.

## 1 Introduction

The global population of people with disabilities is substantial. According to statistics released by the World Health Organization, approximately 1.3 billion people with disabilities were estimated worldwide, among whom over 285 million suffered from visual impairments and the number of individuals with physical disabilities exceeded hundreds of millions. What is more severe is that the scale of this group is being further expanded by the intensification of global population aging and the continuous rise in the prevalence of chronic eye diseases (Manjari et al., 2020). In China, the size of the visually impaired population cannot be ignored either. This large group has been confronted with numerous practical difficulties in daily life, travel and social interaction (Busaeed et al., 2022). However, the existing assistive tools such as white canes and guide dogs, although certain assistance has been provided to some extent, still exhibit significant limitations in adaptability, intelligence and scenario coverage, making it difficult to meet the safe navigation needs of visually impaired people in complex dynamic environments (Pohovey et al., 2025). As an emerging assistive technology, intelligent obstacle avoidance robots have attracted extensive attention in recent years. The design of such robots is intended to simulate the human cognitive process of the external world through the construction of an environmental perception system, so that the identification, positioning and avoidance of dynamic and static obstacles can be realized, thereby providing safe and efficient mobility assistance for people with disabilities (Liu, 2025).

Environmental perception is the foundation and prerequisite for the realization of autonomous navigation in intelligent obstacle avoidance robots. From the perspective of technological evolution, early environmental perception devices mostly relied on simple ultrasonic ranging or infrared sensing, which could only provide contact or short-range non-contact early warnings with single functions and rigid interactive experiences (Ramkumar et al., 2018). At present, however, research has entered an intelligent stage of multi-level data fusion of lidar, RGB-D cameras and ultrasonic sensors. These robots can not only identify obstacles but also understand their specific attributes.

Obstacle recognition and scene understanding constitute the key links for intelligent obstacle avoidance robots to achieve safe navigation. In recent years, object detection algorithms such as the YOLO series have enabled real-time detection and classification, satisfying the stringent requirements of robot systems for low-latency responses. Simultaneous Localization and Mapping technology incorporating semantic information annotation has been developed on the basis of geometric mapping, allowing robots to not only perceive the geometric structure of the environment but also understand its semantic content, for example, distinguishing fixed obstacles from dynamic pedestrians and identifying passable areas from hazardous zones (Hall et al., 2020). The introduction of such high-level semantic information has greatly enhanced the decision-making intelligence of robots in complex social environments (Graf et al., 2024).

Path planning and obstacle avoidance algorithms are the core modules of assistive robots for individuals with disabilities. Global path planning algorithms, such as the A*, Dijkstra, Rapidly-exploring Random Tree (RRT) algorithms and their improved versions, can search for optimal paths under the condition of known maps, ensuring the global optimality of robot movement from the starting point to the target location (Sahoo and Choudhury, 2024). Nevertheless, in dynamic and complex environments, pure global planning struggles to handle sudden obstacles and pedestrian interference. Local path planning algorithms, which evaluate the safety of motion trajectories, have enabled real-time obstacle avoidance for dynamic barriers (Adámek et al., 2022). At present, obstacle avoidance algorithms have evolved from traditional methods to intelligent decision-making driven by deep learning and reinforcement learning, marking a profound transformation of robot obstacle avoidance technology from passive response to active cognition (Waga et al., 2025). Meanwhile, multi-algorithm fusion has become a mainstream paradigm, which not only significantly improves navigation safety but also enhances the efficiency and smoothness of robot movement in dynamic and crowded environments (Le et al., 2024).

In terms of scenario-based applications, intelligent obstacle avoidance robots have advanced from the concept verification stage to the practical and industrialized phases. Assistive navigation systems designed for visually impaired individuals are usually developed in the form of wearable devices, which feature portability and low cost. Compared with traditional white canes, wearable devices can free users’hands and provide more abundant environmental information. For individuals with physical disabilities, intelligent wheelchairs, as typical mobile assistive robot platforms, integrate functions such as autonomous navigation, obstacle avoidance and voice control. The introduction of socially aware navigation technology has enabled intelligent wheelchairs to understand and comply with human social etiquette (Jiang et al., 2024). The multimodal feedback mechanism, which combines voice commands, tactile cues and auditory signals, dynamically adjusts the information presentation mode according to the ambient noise level, ensuring that users with disabilities receive clear and timely navigation guidance in different scenarios (Sridhar et al., 2025).

Based on the above analysis, this paper aims to systematically sort out the research progress of intelligent obstacle avoidance robots for individuals with disabilities, summarize the evolutionary course of environmental perception, obstacle recognition, path planning and scenario-based applications, and provide theoretical support for promoting the construction of an accessible society.

## 2 Environmental perception

Environmental perception serves as the information input interface of intelligent obstacle avoidance robots, whose core task is to convert physical environments into semantic information that is comprehensible and reliable for users with disabilities. Individual sensors include lidar and visual cameras, which are capable of obstacle category recognition, as well as ultrasonic and infrared sensors, which suffer from poor anti-interference performance and low resolution despite their capability for short-range detection.

For the visually impaired group, existing research generally adopts a multi-sensor fusion architecture. For instance, Casado and Pedrino (2025) focused on monocular depth estimation and proposed a novel method based on convolutional neural networks and attention mechanisms, which integrated the convolutional block attention module and a modified global context network, aiming to provide depth information for navigation assistance systems for visually impaired individuals. Ogunsanya and Okoi (2024) described a wearable electronic navigation device integrated with three ultrasonic sensors, one passive infrared sensor, a GPS module, and audio/tactile feedback. In terms of indoor positioning, the inter-row obstacle avoidance weeder designed by Jia et al. (2025) integrated non-contact sensors and mechanical tactile structures to achieve multi-sensor fusion combined with physical contact feedback, thus overcoming the instability of single sensors. Chen et al. (2021) introduced a system that adopted a structured light-based RGB-D camera as the sensor and the control center, and combined SLAM technology with semantic information extracted from the environment. This system ensured that the computing platform could perceive the surrounding environment in real time and feed back information to visually impaired individuals via voice broadcast, as illustrated in Figure 1.

**FIGURE 1:**
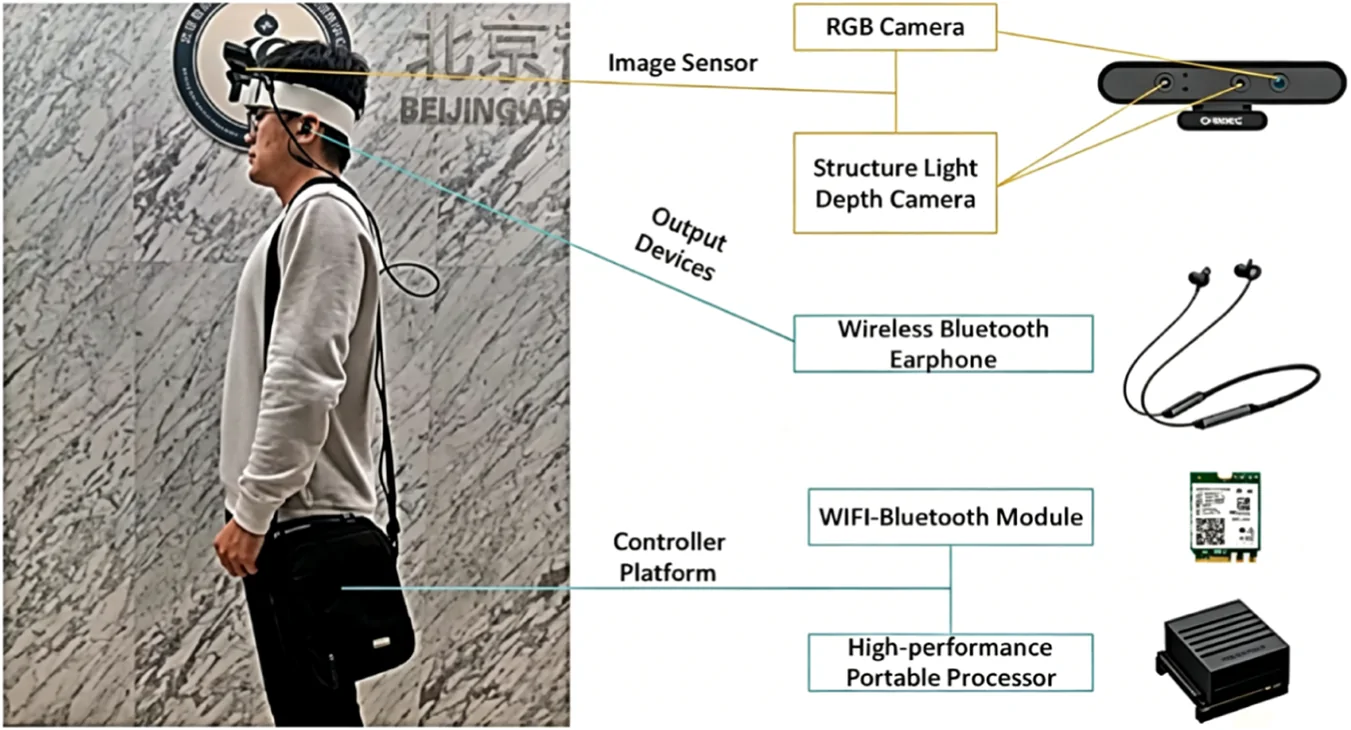
The wearable navigation system platform (Chen et al., 2021).

Aladren et al. (2014) proposed a novel NAVI system based on visual and range information, which utilized consumer-grade RGB-D camera devices and combined depth information with image intensity to realize robust expansion of range-based floor segmentation. Environmental perception is evolving from simple obstacle detection to semantic and scene-based passability analysis. Through multi-sensor fusion, the complexity of travel scenarios for people with disabilities has been effectively addressed, and full-dimensional, high-precision acquisition of environmental information has been achieved. Katzschmann et al. (2018) developed the ALVU system by combining a time-of-flight lidar sensor array with a vibrotactile feedback belt, which was designed to detect low-hanging and high-hanging obstacles and transmit distance-related information. Lu et al. (2021) integrated LiDAR point cloud data with deep reinforcement learning algorithms, and designed a guidance robot for the visually impaired by combining ultra-wideband beacon positioning technology; a LiDAR with a 240° field of view was adopted to perceive environmental states in this system. McCormac et al. (2017) constructed a dense 3D semantic SLAM system by combining the ElasticFusion framework with convolutional neural networks, and real-time semantic segmentation and 3D reconstruction were realized using RGB-D cameras. Wang et al. (2017) developed a vision-based wearable feedback system via the integration of RGB-D cameras with a computer vision feedback system, which could perceive the environment in real time and provide navigation guidance. Mur-Artal et al. (2015) proposed a monocular visual SLAM system based on ORB feature points, and real-time pose estimation was achieved using feature extraction and matching technology, which was applicable to low-cost visual navigation for assistive devices of the visually impaired. The ORB-SLAM series algorithms proposed by Mur-Artal and Tardós (2017) provided a low-cost visual navigation solution for visually impaired assistive devices, and real-time pose estimation and semantic information extraction were realized. Campos et al. (2021) proposed a visual-inertial fusion SLAM system supporting a multi-map framework, which enabled high-precision positioning and navigation for visually impaired individuals in complex environments, while dynamic obstacles were processed and long-term navigation tasks were supported. The YOLO object detection algorithm proposed by Redmon et al. (2016) provided fast obstacle recognition capability for wearable devices of the visually impaired. Said et al. (2023a) developed a lightweight real-time obstacle detection system suitable for embedded devices by adopting the FCOS detector combined with the EfficientNet backbone network and neural architecture search technology. Chen et al. (2023) developed a wearable tactile glove system to transmit obstacle distance information by fusing YOLOv3 object detection, stereo camera disparity ranging and shape memory alloy tactile actuators. In summary, current research is evolving from single-sensor deployment to multi-source sensor fusion. These advances not only improve the robustness of environmental perception through the complementarity of different sensors, but also realize semantic understanding of complex scenarios by leveraging the powerful feature extraction capability of neural networks. These technological progresses provide safer, more reliable and intelligent travel guarantees for visually impaired individuals.

## 3 Obstacle recognition and classification

Obstacle recognition represents the intelligent advancement of environmental perception data, and its technological evolution has undergone a transformation from static geometric detection to dynamic semantic understanding. Early systems identified simple obstacles such as ground protrusions and suspended objects based on rule-based algorithms, which were characterized by high misjudgment rates and incapability of coping with dynamic scenarios. With the infiltration of deep learning technologies, obstacle classification and behavior prediction have become the mainstream.

For visually impaired individuals, recognition results need to be converted into non-visual modal feedback. In terms of unstructured environments, Chang et al. (2025) conducted research on the autonomous obstacle avoidance of mountain tractors, where semantic neural networks were employed to process LiDAR data, extract key semantic information such as the type, size and position of obstacles, and construct 3D maps containing semantic information in combination with laser SLAM technology. Shariatinezhad et al. (2024) focused on the detection of easily overlooked yet safety-critical obstacle categories for blind and visually impaired people, such as edges, slopes and potholes in sidewalk datasets, by utilizing YOLOv5 and transfer learning, and achieved excellent mean average precision on the custom PESID dataset. Wang et al. (2024) proposed the YOLO-OD obstacle detection method; by introducing feature weighting blocks and adaptive bottleneck blocks, the model’s capability of detecting and distinguishing obstacles of varying sizes in cluttered outdoor environments was enhanced, reaching a mean average precision of 30.02% on public datasets, however, there was a lack of uncertainty indicators such as standard deviation or confidence intervals. For indoor navigation, Said et al. (2023a) proposed an obstacle detection system based on the improved YOLOv5 neural network; by integrating DenseNet into the YOLOv5 backbone network and applying channel pruning and quantization techniques, the accuracy and speed of obstacle detection had been significantly improved. In addition, scene recognition is equally important for providing contextual navigation guidance. Said et al. (2023b) proposed an indoor scene recognition system based on Vision Transformer and dual multi-scale attention to help BVIP understand the surrounding environment. Jiang et al. (2025) proposed an image adaptive enhancement-based algorithm for target obstacle detection and avoidance in port area environments; by introducing hyperparameter optimization and multi-scale detail enhancement modules, the recognition accuracy of obstacles in complex environments was improved. Omer et al. (2025) explored the integration of human feedback into the control loop of mobile robots using the electroencephalogram brain-computer interface method for real-time obstacle detection and avoidance. Passive BCI modified from the original indirect robot control, integrating ErrPs to detect user-perceived errors during robot navigation, as illustrated in Figure 2. Active BCI modified from the original direct robot control, incorporating SSVEPs to enable active user control during robot navigation, as illustrated in Figure 3.

**FIGURE 2:**
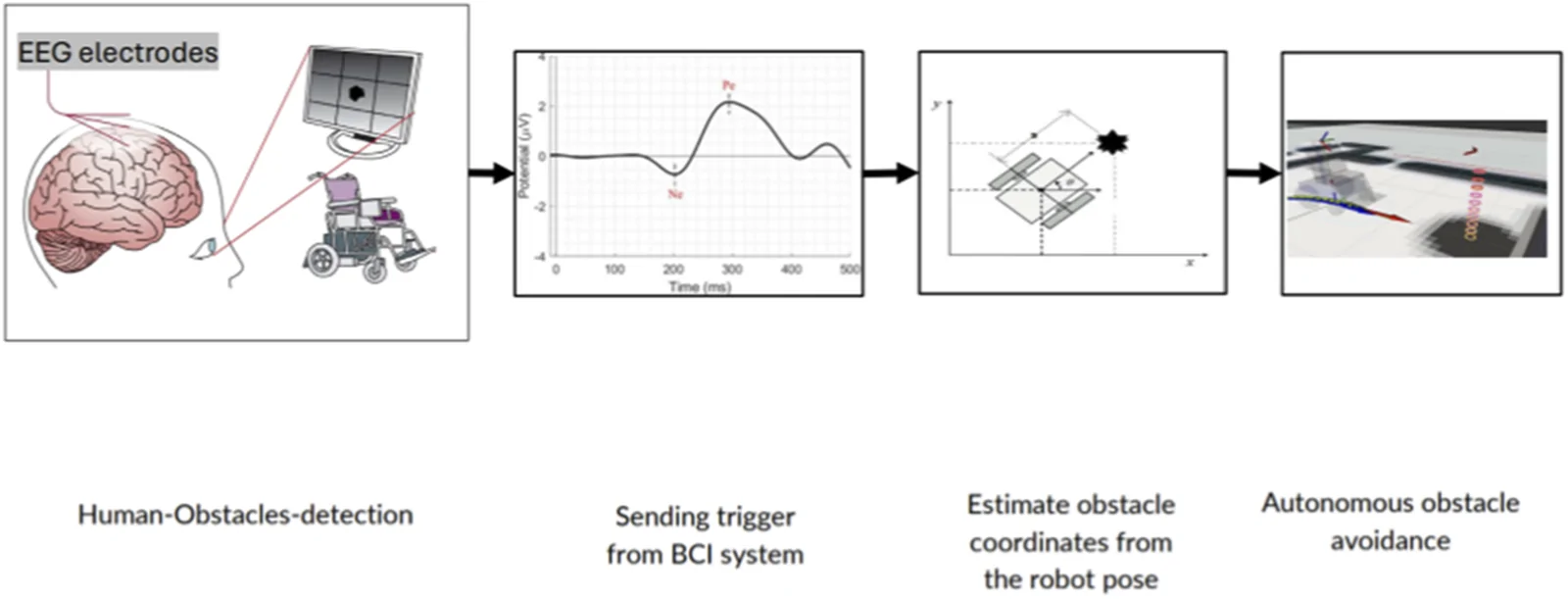
Passive BCI (Omer et al., 2025).

**FIGURE 3:**
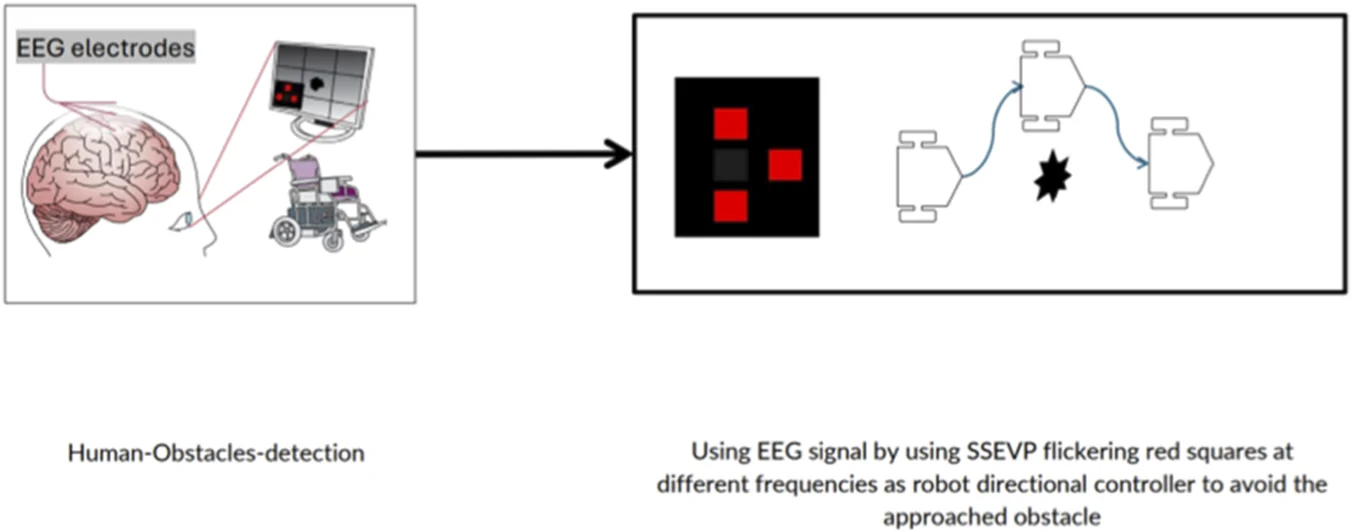
Active BCI (Omer et al., 2025).

Ahmed and Das (2025) compared the multi-category detection performance of YOLOv3, YOLOv5 and Faster R-CNN on the KITTI dataset, and it was verified that Faster R-CNN achieved higher accuracy in pedestrian and cyclist detection, while YOLOv5 attained a better trade-off between real-time performance and accuracy, however, uncertainty indicators such as confidence intervals, standard deviations, or repeated trial counts had not been thoroughly analyzed. Maurya and Verma (2024) adopted deep convolutional neural networks optimized by social optimization algorithms combined with generative adversarial networks for data augmentation, and extracted CNN features, local binary texture features of shapes and hierarchical skeleton features to achieve high-precision classification of multi-category obstacles. Alarood et al. (2025) proposed the BViT-CKNN architecture, which combined the bilateral filtering Vision Transformer with the convolutional k-nearest neighbor module, and high-precision discrimination of multi-category obstacles across 80 categories on the COCO dataset was realized. Ali (2024) compared the performance of CNN and Transformer models in visually impaired navigation, and found that YOLOv8x outperformed DETR after fine-tuning, but there was a lack of relevant statistical variance or experimental uncertainty indicators. Bayat et al. (2023) customized a Vision Transformer for environmental detection and collision prediction, and the self-attention mechanism was utilized to model global contextual relationships, thus enabling accurate recognition of multi-category obstacles in complex outdoor environments. An et al. (2026) proposed a few-shot segmentation framework with a negative contrastive learning branch, which realized precise classification of passable areas and multi-category obstacles with only 1–5 labeled samples. Mu et al. (2023) employed a fast instance segmentation network combined with multi-scale 2D convolutional units to capture local and global contextual features, thereby distinguishing obstacles of different instances. Ga et al. (2025) proposed a wearable obstacle avoidance device based on cross modal learning, which achieves the detection and classification of multi class obstacles. A 7 month field test conducted by 12 visually impaired volunteers showed that the device achieved a 100% collision avoidance rate in various indoor and outdoor scenarios. Two factor analysis of variance confirmed its significant advantage over traditional white rattan. Aladren et al. (2014) proposed a floor segmentation-based semantic navigation method to detect and classify the main structural elements of scenes, realizing semantic-level recognition of multi-category environmental elements in indoor scenarios. Shi and Rajkumar (2020) proposed the Point-GNN graph neural network, which predicted the category and shape of the object to which each vertex belonged via graph convolution, and leading accuracy in multi-category 3D object detection was achieved using pure point cloud data on the KITTI benchmark. Zhang et al. (2021) captured the inter-point relationships through a point cloud completion module combined with a relational graph neural network, realizing precise classification and localization of multi-category 3D obstacles. However, a prominent contradiction exists between the real-time constraints in dynamic environments and the limited edge computing resources. Maintaining recognition accuracy while ensuring frame rate, as well as balancing model complexity and energy consumption, remains the core issue in algorithm optimization.

## 4 Path planning and obstacle avoidance algorithms

Path planning and obstacle avoidance planning are the core modules for the autonomous navigation of robots. According to the completeness of environmental information and the time scale of planning, four major strategies have been formed in this field, namely, global planning, local planning, deep reinforcement learning, and multi-algorithm fusion, as illustrated in Figure 4. Among these strategies, global planning algorithms include A*, Dijkstra, and RRT algorithms, which rely on pre-acquired complete environmental maps to search for optimal paths in static spaces. Local planning algorithms, including the Dynamic Window Approach (DWA) and the artificial potential field method, rely on real-time environmental information perceived by sensors to perform online obstacle avoidance adjustments in dynamic or unknown environments (Bai et al., 2021).

**FIGURE 4:**
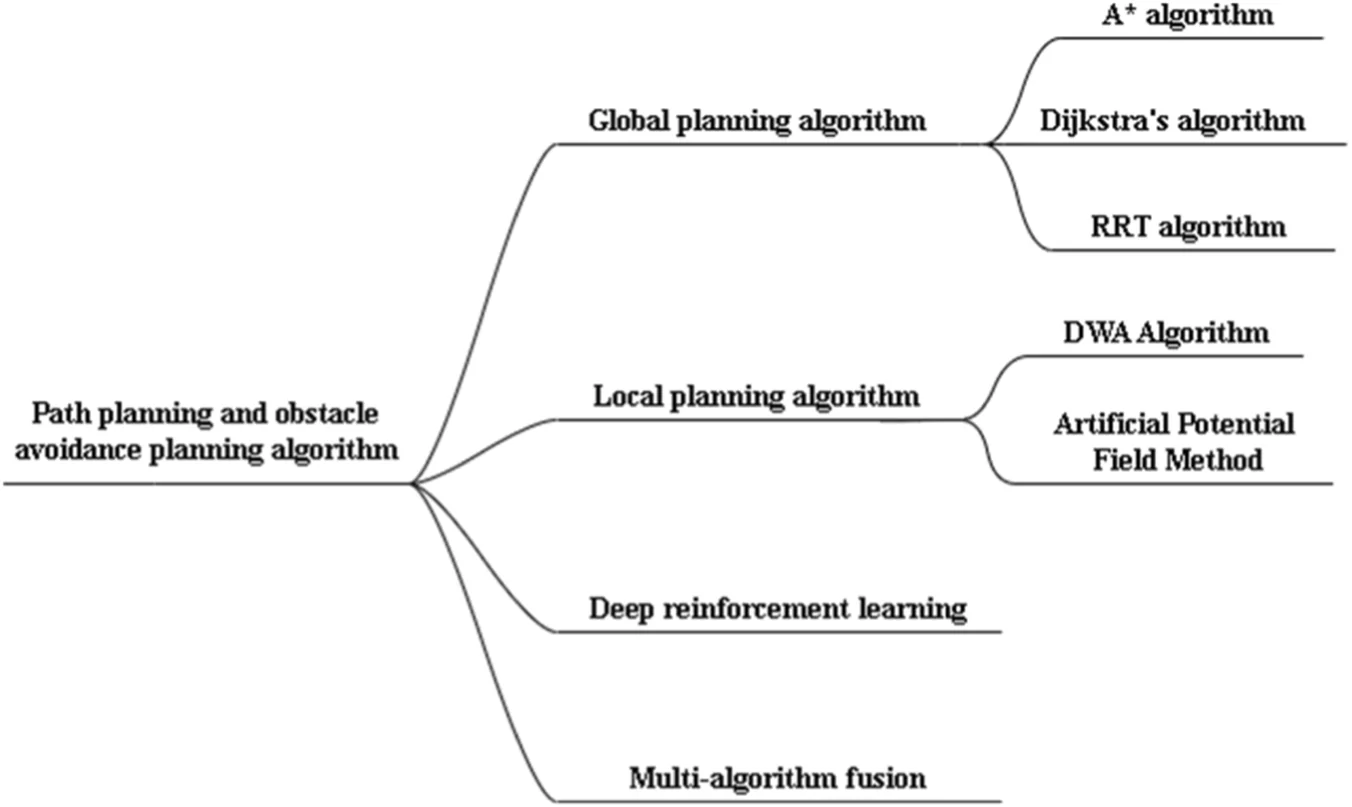
Classification of path planning and obstacle avoidance algorithms.

### 4.1 Global path planning algorithms

Global path planning algorithms are based on complete environmental map information, and the optimal or feasible path from the starting point to the destination is calculated during the planning phase (Tang Y. et al., 2025). Such algorithms usually assume that the environment is static and known, and their core objective is to search for a trajectory within the global scope that satisfies the requirements of shortest path, minimum energy consumption, maximum safety, etc (Xu and Zhang, 2025). Commonly used algorithms include the Dijkstra algorithm, the A* algorithm and their improved versions.

#### 4.1.1 Dijkstra algorithm

The Dijkstra algorithm was proposed by the Dutch computer scientist Edsger W. Dijkstra in 1959. It is applied to find the shortest path from a source node to all other nodes in a weighted graph. The algorithm boasts the advantages of high efficiency, simplicity, intuitiveness and asymptotic optimality, but it has drawbacks such as difficulty in handling negative-weight edges, high spatial requirements, and unsuitability for real-time updates (Ugwoke et al., 2025). Alshammrei et al. (2022) modeled the mixed reality (MR) barrier-free environment as a graph consisting of nodes, edges and weights based on the improved Dijkstra algorithm. Then, the Dijkstra algorithm was used offline to generate the shortest path that drives the MR system from the starting point to the target point. As a result, the robot was made to follow the pre-obtained path during movement and stop at each node to detect whether obstacles exist between the current node and the subsequent node, as illustrated in Figure 5.

**FIGURE 5:**
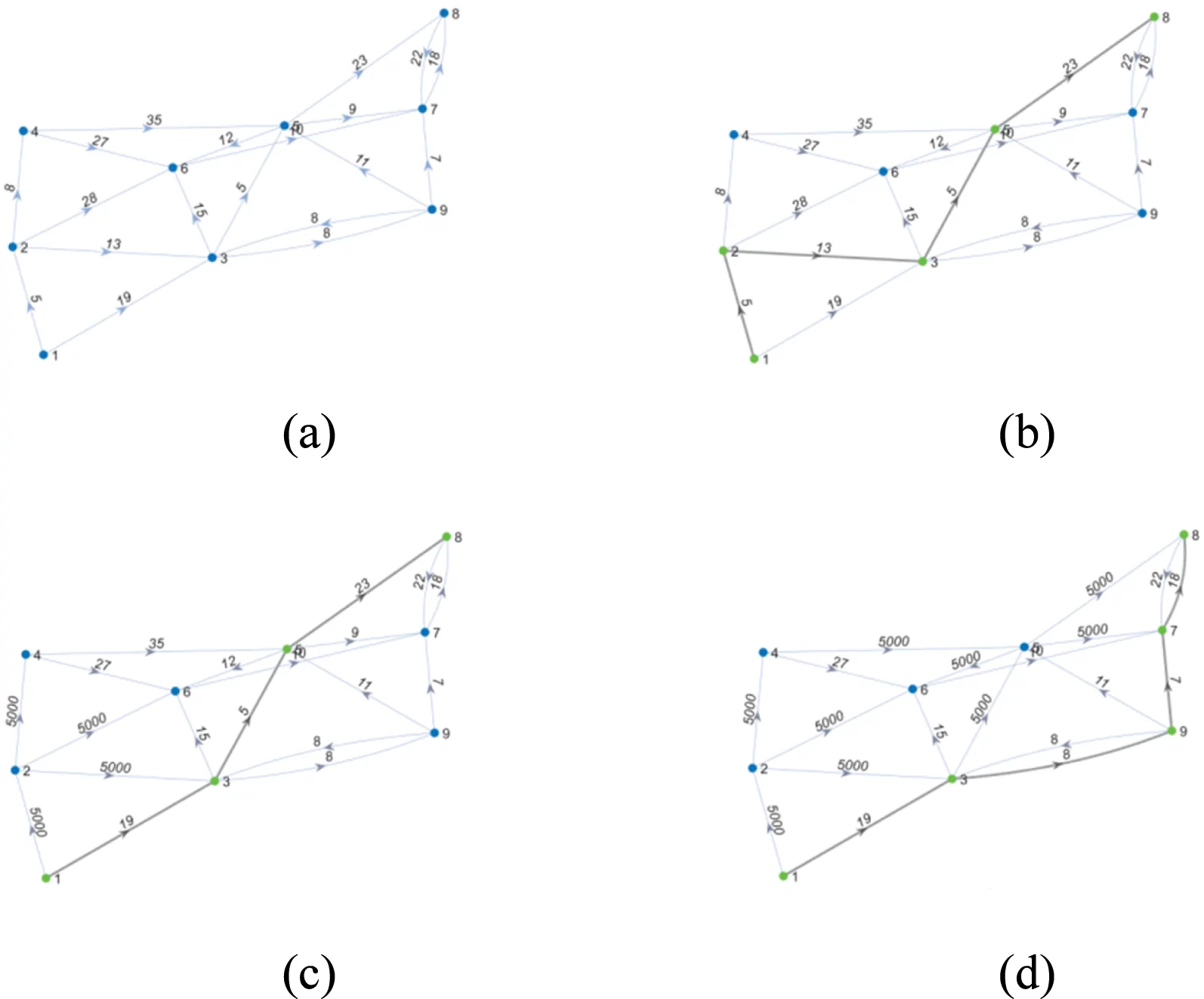
Illustration of the diagraphs of the path planning and obstacle avoidance implementation (Alshammrei et al., 2022). **(a)** Initial weighted directed graph of path planning environment. **(b)** Initial optimal path under barrier free conditions. **(c)** Re planned path after excluding node 2. **(d)** Final re planned path after excluding nodes 2 and 5.

Luo et al. (2020) proposed an extended Dijkstra algorithm to address the challenge of optimal path planning on curved surfaces. A 2D developable passable channel of the curved surface was constructed, and the optimal path was solved on this channel. The global shortest path was obtained by traversing all 2D developable passable paths, and then the optimal path on the curved surface was derived through inverse transformation. Guo et al. (2017) proposed was an improved Dijkstra algorithm, which can find all equidistant shortest paths in a rectangular environmental map. By adding running time as an evaluation index for path planning, a path with the shortest distance and optimal time was obtained. Bing and Lai (2022) put forward two optimization and improvement schemes for the Dijkstra algorithm. The first was to modify the calculation method of the algorithm to make it more directional, thereby reducing the total amount of computation. The second was to reduce the number of nodes through a graphical model to optimize the calculation process. By comparing the calculation results and complexity with those of the traditional algorithm, the effectiveness of the improved methods was verified, the algorithm complexity was reduced, and the actual work efficiency was improved.

Costa et al. (2025) systematically reviewed the application of monotonic priority queues in the optimization of the Dijkstra algorithm, and pointed out that the characteristic of monotonic priority queues, where the minimum key value does not decrease with time, makes them particularly suitable for label-setting algorithms such as Dijkstra. Yu and Saniie (2025) developed the VISA system, which adopted the Dijkstra algorithm for path planning based on node maps and combined it with ArUco marker recognition and positioning. The shortest path from the current position of visually impaired users to the target node was calculated by the Dijkstra algorithm. Kostavelis et al. (2016) used the Dijkstra algorithm for global path planning on semantic topological graphs based on the COLD dataset, providing navigation services based on semantic information for visually impaired users. Viswanathan et al. (2024) constructed a nested graph structure at the target layer, pose layer and component layer, and adopted the Dijkstra algorithm to plan paths on the local graph of each layer, realizing hierarchical navigation based on semantic queries for visually impaired assistive robots. Shi et al. (2022) adopted the Dijkstra algorithm combined with the state lattice algorithm for global path planning in the intelligent parking scenario, ensuring the global optimality of the path. Calderita et al. (2020) compared the performance of the classic Dijkstra planner and the socially aware planner in nursing home environments. The Dijkstra algorithm plans the shortest path but invades personal space, while the improved socially aware path planning ensures safety while maintaining distance, providing a benchmark for socially aware navigation of assistive robots for visually impaired elderly people.

#### 4.1.2 A*(A-star) algorithm

The A* algorithm is a heuristic search algorithm used to find the shortest path from the initial node to the target node in a graph. It uses a heuristic function f(n) = g(n)+h(n) to determine the search order, where g(n) represents the actual path cost from the starting point to any node n. Every time a new node is reached from the starting point through a path, g(n) is accumulated with the movement cost to that node. h(n) denotes the estimated cost from the node to the target. For two points in a grid, the Euclidean distance is usually adopted as the heuristic function of the algorithm. f(n) represents the estimated total cost of the node (Xuan et al., 2025). This algorithm has the advantages of optimality, high efficiency and scalability (Fang et al., 2025), but it suffers from drawbacks such as insufficient grid map modeling, inadequate selection of neighboring nodes, inability to adaptively meet the requirements of different tasks, and low computational efficiency for large-scale maps. Liu and Liu (2025) introduced adaptive weights into the heuristic function of the A* algorithm, thereby reducing the number of redundant nodes and the required computation time, as illustrated in Figures 6, 7.

**FIGURE 6:**
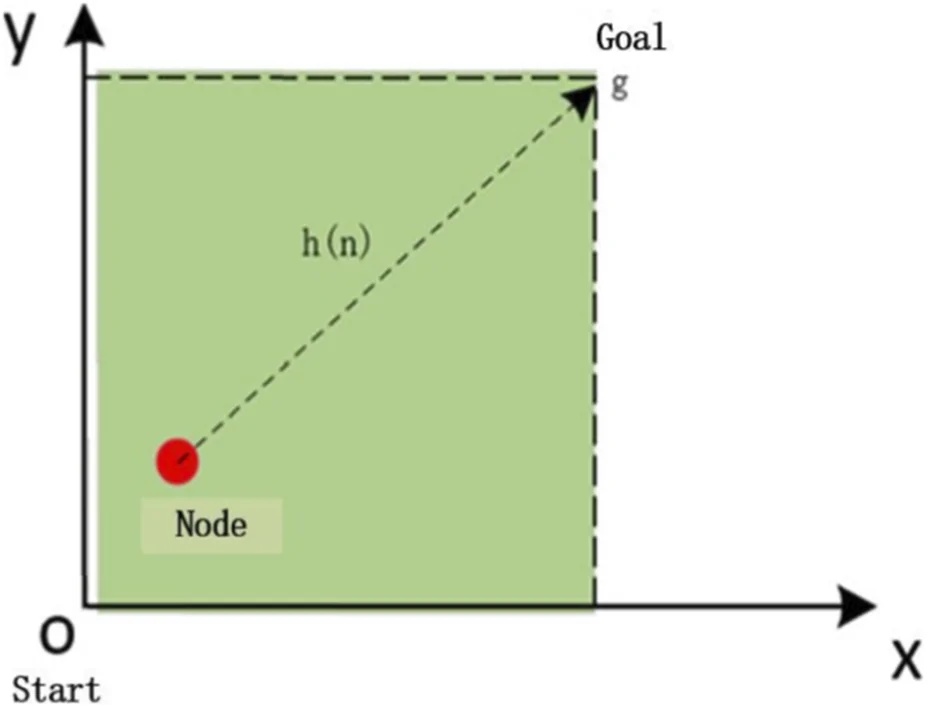
Traditional A* algorithm search range (Liu and Liu, 2025).

**FIGURE 7:**
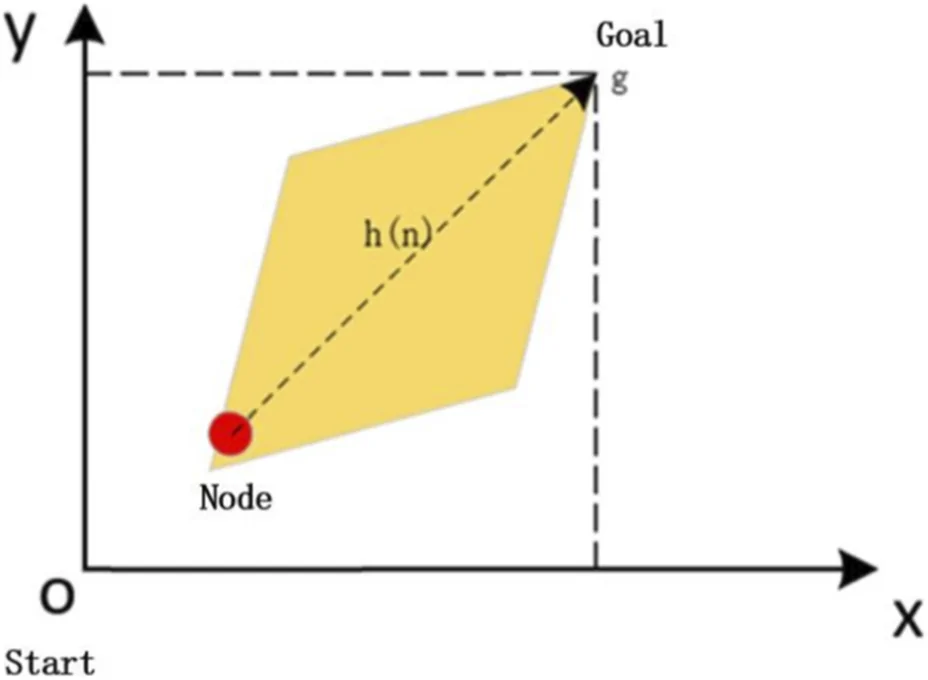
Improved search range of A* algorithm (Liu and Liu, 2025).

Liu et al. (2025a) proposed an enhanced A* pathfinding algorithm. By dividing the path planning process into different phases, the search efficiency and accuracy were improved, and unnecessary node exploration was thus reduced. Huang et al. (2025) proposed an improved A* algorithm. To address the problems of low search efficiency in 16-neighborhood search and unsmooth paths in 8-neighborhood search, a novel 5-neighborhood search method was introduced. This method retained the path smoothness characteristic of 16-neighborhood search while achieving higher time efficiency, as illustrated in Figure 8.

**FIGURE 8:**
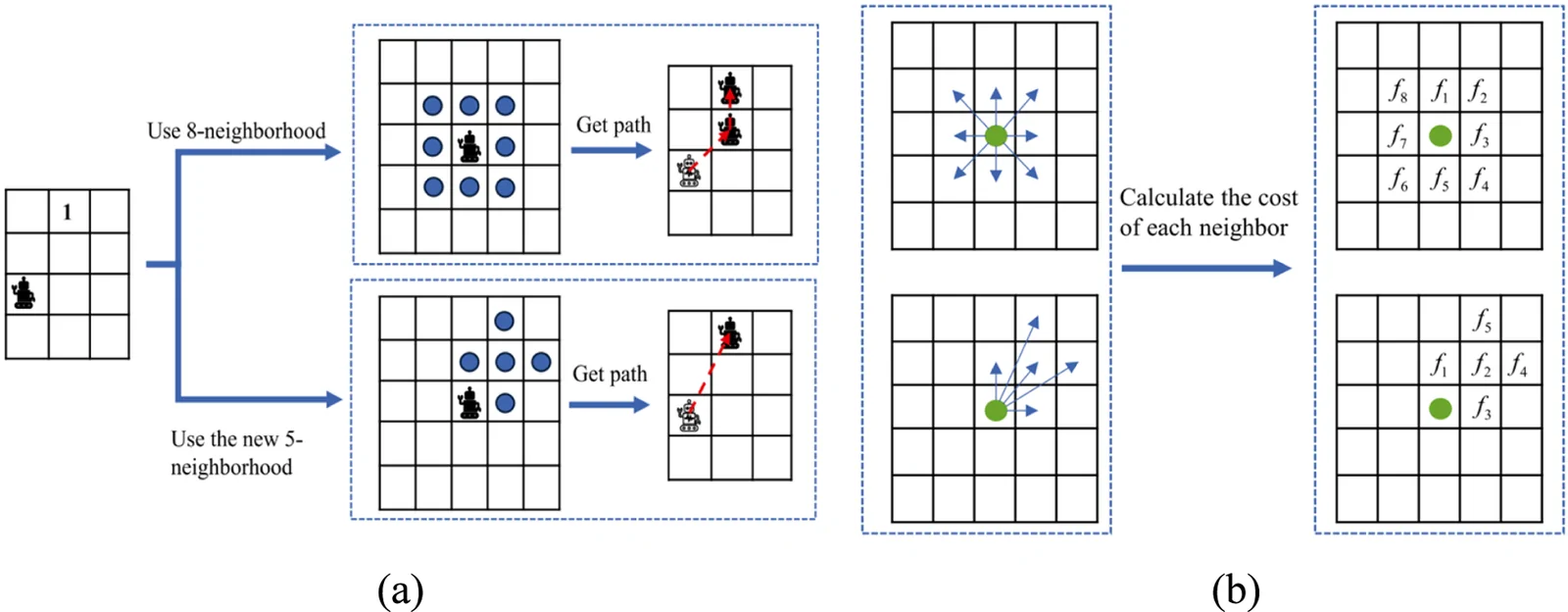
The function of the new 5-neighborhood search: **(a)** making path smother and shorter; **(b)** enhancing the search efficiency (Huang et al., 2025).

Xu et al. (2024) put forward an improved A* algorithm, the octagonal neighborhood was replaced with obstacle-free rectangular boundary elements, and map exploration was performed bidirectionally from both the starting point and the target point, resulting in fewer generated nodes. Hart et al. (1968) proposed the A* algorithm by introducing a heuristic function on the basis of the Dijkstra algorithm. The admissible heuristic function h(n) was used to guide the search direction, which ensured the acquisition of the optimal path while improving search efficiency. El–E et al. (2024) achieved a significant reduction in travel time in a simulated urban environment in Berlin by incorporating real-time traffic and weather data into the heuristic function of the A * algorithm and transforming it into a deep learning model. Pal et al. (2012) improved the A* algorithm by combining neural networks with genetic algorithms, constructed a neural network model for environmental information, and established the relationship between obstacle avoidance paths and model outputs. Two-dimensional path point encoding was converted into one-dimensional encoding, and the fitness of obstacle avoidance paths was integrated with that of the shortest distance. Kavraki et al. (2002) adopted the Probabilistic Roadmap (PRM) algorithm combined with A* graph search, randomly sampled nodes in the high-dimensional configuration space, and used the A* algorithm to search for the optimal path on the constructed graph. This approach provided high-dimensional motion planning capabilities for wheelchair navigation of visually impaired individuals, solving the complex path planning problem of multi-degree-of-freedom assistive devices. Viswana et al. (2024) constructed a nested graph structure at the target layer, pose layer and component layer, and adopted the A* algorithm to plan paths on the local graph of each layer, realizing hierarchical navigation based on semantic queries for visually impaired assistive robots.

#### 4.1.3 Rapidly-exploring random tree (RRT) algorithm

The RRT algorithm starts with the initial point as the root node, randomly samples points in the state space, and guides the tree to grow toward the sampled points to gradually cover the entire space until the target region is reached (Otte and Frazzoli, 2016). Due to its strong probabilistic completeness and excellent search capability, the RRT algorithm has been extensively studied. However, owing to its inherent randomness, it has limitations such as low planning efficiency, suboptimal path quality, and inconsistent paths generated when the same task is executed repeatedly. For this reason, numerous studies have been dedicated to the improvement of the classic algorithm. To address the problems of high randomness, excessive redundant nodes, and low search efficiency in the original RRT-Connect algorithm, Xu et al. (2025) proposed a path planning method combining RRT-Connect with Informed RRT. Compared with the original RRT-Connect algorithm, the initial solution time, total number of nodes, and initial path cost were reduced by more than 11%, 8.5%, and 2.5%, respectively. Nasir et al. (2013) proposed the RRT*-Smart algorithm, which accelerated the convergence speed of RRT through two new technologies including path optimization and intelligent sampling, so as to achieve the optimal or near-optimal solution more quickly and reduce the execution time. Furthermore, Chiang et al. (2019) proposed the RL-RRT algorithm. This method used the reachability estimator learned from the reinforcement learning (RL) strategy to guide motion planning under dynamic constraints, which effectively improved the efficiency of path generation satisfying the system dynamic constraints, as illustrated in Figure 9.

**FIGURE 9:**
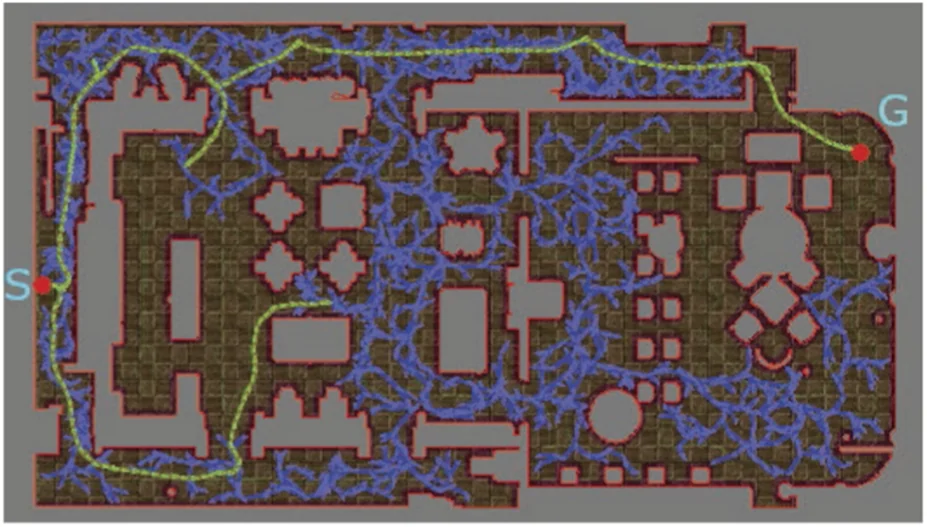
Example trees constructed with RL-RRT (yellow) and SST (blue) for a kinodynamic car navigating from start (S) to goal (G) (Chiang et al., 2019).

Wang and Sanfelice (2024) proposed the HyRRT-Connect algorithm, a bidirectional rapidly-exploring random tree motion planning algorithm for hybrid dynamic systems. To address the possible discontinuity between the results of forward and backward propagation, the backward part of the motion plan was reconstructed via forward hybrid time simulation, which effectively eliminated the discontinuity. Salzman and Halperin (2016) proposed the asymptotically near-optimal RRT algorithm for fast and high-quality motion planning. This algorithm achieved continuous interpolation between RRT and RRT*, balancing computational speed and path quality. Webb and van den Berg (2012) proposed the Kinodynamic RRT algorithm to realize optimal motion planning for systems with linear differential constraints, ensuring that the generated paths satisfy the kinematic constraints of robots and thus guaranteeing the feasibility and safety of the planned paths in practical execution. Tengesdal et al. (2025) conducted the first comparative study on the performance of PQ-RRT, Informed RRT, RRT* and RRT under nonholonomic constraints. The results demonstrated that although the optimal RRT variants could generate paths closer to the distance-optimal solution, they required more computation time. In summary, the improvements of the RRT algorithm have been carried out along multiple dimensions including efficiency enhancement, optimality guarantee, integration of intelligent guidance, and adaptation to complex systems, laying a solid foundation for the research and development of intelligent assistive devices in the future.

### 4.2 Local dynamic obstacle avoidance algorithms

Different from global path planning algorithms that rely on complete environmental maps, local path planning algorithms are based on real-time perception information of robots, performing path planning and obstacle avoidance decision-making within a short time and short distance to achieve real-time response to dynamic environments (Claro, 2025). Commonly used local dynamic obstacle avoidance algorithms mainly include the Dynamic Window Approach (DWA) and the artificial potential field method.

#### 4.2.1 Dynamic window approach (DWA)

The DWA algorithm performs multiple sets of sampling in the velocity space of the robot, predicts future motion trajectories based on the current state, and selects the optimal path through an evaluation function. This method boasts high computational efficiency and can meet the requirements of real-time obstacle avoidance. Moreover, it can take into account dynamic constraints such as the maximum acceleration and angular velocity of the robot, making the planned path more consistent with the actual motion capability. However, it has drawbacks such as delayed obstacle avoidance, frequent direction changes, time-consuming planning and parameter sensitivity (Vošahlík and Obitko, 2025). Seo et al. (2026) proposed a hybrid path planning framework combining large language models with DWA, namely, LLM-DWA. This method utilizes the reasoning capability of LLMs to interpret environmental prior information and generate appropriate relay waypoints to guide the robot out of predicaments. Wang et al. (2023) proposed an adaptive obstacle avoidance algorithm named DDPG-DWA, which combines Deep Deterministic Policy Gradient (DDPG) with DWA. The obstacle avoidance problem was converted into an optimal learning incentive problem, and DDPG was utilized to realize self-learning of obstacle avoidance strategies. An experience replay mechanism was adopted to optimize the trajectory evaluation function of DWA, which effectively circumvented the defect of traditional DWA falling into local optimal solutions in complex environments and solved the problem of action output within continuous velocity and steering angle intervals, as illustrated in Figure 10.

**FIGURE 10:**
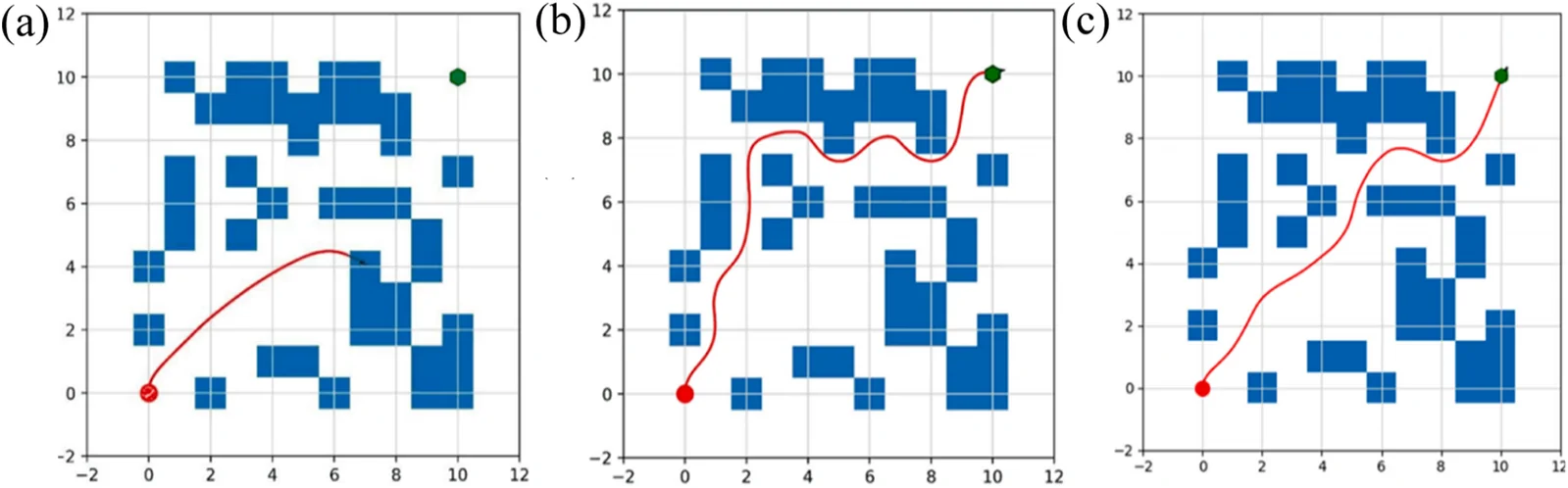
The trajectory of the DDPG-DWA algorithm at different iteration stages (Wang et al., 2023). **(a)** DDPG-DWA (1). **(b)** DDPG-DWA (2). **(c)** DDPG-DWA (3).

Szczepański (2025) proposed to adopt the Pattern Search optimization algorithm to reduce the computational demand of DWA. The results demonstrated that this method reduced the computational load by nearly half while achieving a similar resolution of control signals. Betoño et al. (2025) proposed an enhanced Predictive Dynamic Window Approach. The results showed that compared with the traditional P-DWA, this method reduced energy consumption by approximately 9% on average while maintaining high computational performance, with the iteration time ranging from 15.6 ms to 18.5 ms. Chen H. et al. (2025) proposed a DT-DWA algorithm based on a decision tree classifier. The decision tree classifier was integrated into DWA, and the features affecting decision-making were considered to realize adaptive adjustment of weights. Fox et al. (2002) proposed the Dynamic Window Approach, which performs sampling in the velocity space (v,w). Combined with the robot’s kinematic constraints and obstacle information, the optimal velocity command was selected through an evaluation function to achieve real-time local obstacle avoidance. Tafnakaji et al. (2023) proposed the Dynamic Window Approach, which performs sampling in the velocity space (v,w). Combined with the robot’s kinematic constraints and obstacle information, the optimal velocity command was selected through an evaluation function to achieve real-time local obstacle avoidance, providing a basic velocity space planning framework for visually impaired assistive robots. Tafnakaji et al. (Benayed and Masmoudi, 2026) adopted the DWA algorithm for local path planning in intelligent wheelchairs, realizing dynamic adjustment of the safety radius. The obstacle avoidance success rate reached 95%, and the replanning delay was less than 1 s. Li et al. (2017) implemented DWA local obstacle avoidance on a low-cost wheelchair platform, combined with Gmapping for mapping and Adaptive Monte Carlo Localization for positioning, providing indoor autonomous exploration and navigation capabilities for visually impaired users. Cui et al. (2024) integrated the DWA algorithm into intelligent wheelchairs for local path planning, combined with multimodal human-computer interaction. In summary, the improvements of the DWA algorithm have been carried out along multiple paths. These studies have jointly promoted the enhancement of DWA’s navigation capability in complex, dynamic and multi-constraint environments, laying a solid technical foundation for the construction of safe, efficient and comfortable assistive robots for people with disabilities.

#### 4.2.2 Artificial potential field method (APF)

The APF algorithm regards the target point as an attractive source and obstacles as repulsive sources, such that the robot moves toward the target and avoids obstacles under the action of resultant force. This method features simple principles, low computational complexity, good real-time performance and smooth paths, but it has inherent defects such as local minima, target inaccessibility and oscillation (Pham et al., 2026). To address these problems, researchers have proposed various improvement strategies. He et al. (2025) proposed an optimized path planning method based on improved APF, which effectively overcame the problems of target inaccessibility and local minima of the traditional APF algorithm. Zhu et al. (2026) proposed an enhanced APF obstacle avoidance method. The APF method for route planning guidance was improved, a route-based target recovery mechanism was developed, and a yaw recovery force was introduced to ensure that ships could safely and timely return to the planned route after successfully avoiding other ships. Zhang et al. (2024) proposed an optimized APF method. By introducing a distance adjustment factor to correct the defects of traditional APF, a safe distance model and a sub-target virtual potential field were established to generate collision-free paths. Xi et al. (2025) proposed an innovative algorithm integrating improved Probabilistic Roadmap and APF, the inherent defects of APF were effectively solved. Zhao et al. (2025) proposed an upper-layer obstacle avoidance strategy integrating Model Predictive Control and improved APF, multi-objective obstacle avoidance and trajectory tracking control coupled with MPC and APF were realized. Khatib (1986) proposed the artificial potential field method, constructing a virtual potential field to guide robot movement. The robot was regarded as a point in the potential field, subjected to the attractive force from the target point and the repulsive force from obstacles. Real-time obstacle avoidance was realized through the gradient descent method. Hwang and Ahuja (1992) systematically expounded the application of the potential field method in path planning, established the mathematical framework of the potential field method, and provided a local path planning method with theoretical foundations for visually impaired navigation devices. Sharma (2024) developed a quadruped robot-guided wheelchair system, connected by a virtual flexible traction rope, and constructed a dynamic protection polygon. The quadruped robot uses APF for obstacle avoidance, and the wheelchair travels along an obstacle-free path, providing dual-robot collaborative navigation for visually impaired individuals. Erturk et al. (2024) compared the VFH, APF and DWA algorithms, and selected APF as the core of local obstacle avoidance to develop a wheelchair-assisted driving system. APF calculates virtual repulsive force to achieve real-time obstacle avoidance, which performs more stably in narrow channels than VFH and has higher computational efficiency than DWA. Baziyad et al. (2025) proposed the Bulldozer technology, which systematically identifies and eliminates local minima regions through a backfilling mechanism, constructs a pre-computed potential field, detects local minima in blocks, iteratively increases the potential value to eliminate traps, and introduces a slope mechanism to help robots escape from traps, providing reliable navigation without local minima for visually impaired assistive robots. Masehian and Amin‐Naseri (2004) combined Voronoi diagrams, visibility graphs and potential field methods to solve path planning problems, providing parameter trade-offs between safety and path length. The generated paths are shorter than those of pure Voronoi diagrams and potential field methods, and faster than those of visibility graphs. Xiao et al. (2020) used Bayesian change point detection to segment expert demonstrations into coherent contexts, and found the optimal parameter set through behavioral cloning, allowing APF parameters to be dynamically adjusted according to contexts during operation to improve planning performance.

### 4.3 Deep reinforcement learning (DRL)

DRL has become the key to improving the autonomy and adaptability of robots. Faced with dynamic, unknown and energy-constrained environments, traditional path planning algorithms have limitations in practical applications (Tang S. et al., 2025). In contrast, DRL enables robots to directly learn optimal obstacle avoidance strategies from interactions with the environment through an end-to-end learning approach. Lu et al. (2021) defined the state space as a combination of LiDAR point cloud data and the relative position of the target point. They used LiDAR scanning data with a 240° field of view and a resolution of 1°, and stacked 4 consecutive frames of distance data and 10 frames of relative position of the target point as a single observation space. The action space consists of normalized linear velocity and angular velocity, with linear velocity limited to [0,1] and angular velocity limited to [−1,1]. The upper limit of linear velocity is $v_{\max}=1m/s$ , and the upper limit of angular velocity is $w_{\max}=1\text{rad}/s$ . In addition, the system adopts an action soft update mechanism to smooth the angular velocity output:

$$\omega_{t}=\lambda \omega_{t}+\left(1-\lambda \right)\omega_{t-1}$$

Among them, λ is the updated parameter, which effectively reduces the frequent jumping of angular velocity and improves the user’s ride comfort. The reward function adopts a mixture of sparse and dense design.

$$r=r_{\text{goal}}+r_{\text{reach}}+r_{\text{collision}}$$

$$r_{\text{goal}}=\left\{\begin{array}{cc} 0.2 & \text{if toward the goal}\\ -0.2 & \text{else} \end{array}\right.$$

$$r_{\text{reach}}=\left\{\begin{array}{ll} 100 & \text{if reach the goal}\\ 0 & \text{else} \end{array}\right.$$

$$r_{\text{collision}}=\left\{\begin{array}{ll} -10 & \text{if collision}\\ 0 & \text{else} \end{array}\right.$$

Continuously guiding the robot towards the target through $r_{\text{goal}}$ , providing sparse positive rewards to reach the target through $r_{\text{reach}}$ , and imposing significant negative penalties on collisions through $r_{\text{collision}}$ . Although the reward function itself does not explicitly penalize the rate of change in angular velocity, the system adds a “soft update” post-processing module $\left(\omega_{t}=\lambda \omega_{t}+\left(1-\lambda \right)\omega_{t-1}\right)$ after the policy output, which significantly suppresses frequent jumps in angular velocity through exponential smoothing over time and indirectly constrains the behavior of sudden turns.

Gao et al. (2025) proposed an attention-enhanced Dueling DQN, designing an energy efficiency-centered multi-objective reward function to realize the source generation of optimal low-energy trajectories in three-dimensional space. Sun et al. (2025) developed a multi-level Proximal Policy Optimization framework integrating visual perception and hierarchical motion planning. RGB-D images were processed by YOLOv10 to construct dynamic 3D occupancy maps, which were combined with RRT path search and B-spline trajectory smoothing to realize a closed loop of end-to-end perception-decision-execution. High success rates were achieved in dynamic obstacle scenarios, as illustrated in Figure 11.

**FIGURE 11:**
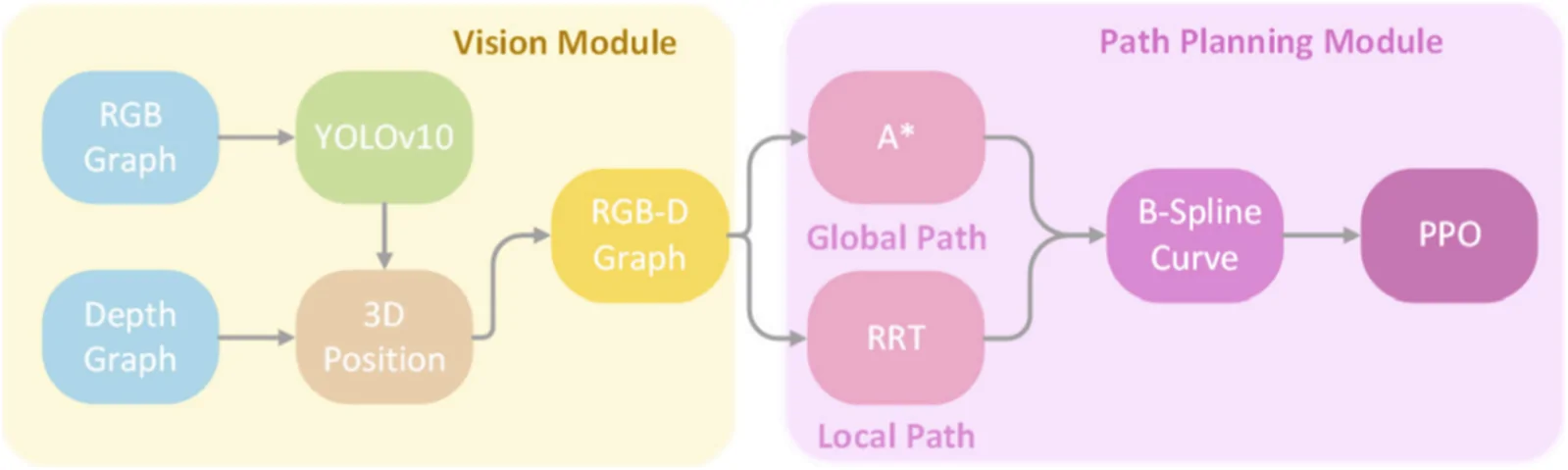
The workflflow of the path planning system (Sun et al., 2025).

However, pure reinforcement learning methods suffer from problems such as unstable training and low sample efficiency. Therefore, numerous studies have shifted toward hybrid architectures that integrate traditional planning methods with DRL. Wang et al. (2025) developed a deep reinforcement learning framework based on the Soft Actor-Critic algorithm. To address the limited adaptability of existing transfer learning methods, an action-level fusion mechanism was proposed, which dynamically integrates prior knowledge and current strategies during the inference process, thereby achieving more flexible knowledge transfer. Liu et al. (2025b) integrated deep reinforcement learning with PID control. By simplifying the task space, the collaborative challenges of dynamic target tracking and multi-obstacle avoidance for robots were effectively solved. Huang et al. (2023) constructed a reinforcement learning model for null-space obstacle avoidance, where the state and reward function of the reinforcement learning agent are independent of specific obstacle information, enabling it to adapt to dynamic environmental changes. Schneider et al. (2025) proposed a novel reinforcement learning framework that combines the modified APF method with the Deep Deterministic Policy Gradient algorithm. This approach effectively mitigates the inherent limitations of APF in obstacle-dense environments and improves performance in both simple and complex scenarios. Mnih et al. (2015) proposed the Deep Q-Network, which combines deep learning with Q-learning. A neural network was used to approximate the Q-value function, and training stability was ensured through the experience replay mechanism and target network. Human-level control was achieved in games, providing decision-making capabilities for visually impaired assistive robots in high-dimensional state spaces. Van Hasselt et al. (2016) proposed Double DQN, which solves the Q-value overestimation problem by decoupling action selection and evaluation. More stable Q-value estimation is realized in complex environments, providing more accurate obstacle avoidance decisions for visually impaired navigation devices. Wang et al. (2016) proposed Dueling DQN, which decomposes the Q-value into state value and action advantage. The network structure was improved to enable the agent to focus more on state value. Doshi et al. (2025) adopted Double DQN to train quadrotor UAVs for obstacle avoidance. Obstacle avoidance strategies were learned through depth image input in a custom “Wobbles” training field. Farkh et al. (2025) adopted a hybrid framework of DQN and large language models to realize real-time decision-making on STM32 microcontrollers. Navigation strategies were optimized by LLMs. AlMahamid and Grolinger (2025) proposed Agile DQN, which combines a deep recurrent attention model and adopts a multi-glance strategy to dynamically focus on key regions of the state space. Convergence is accelerated through experience replay, providing obstacle avoidance capabilities in complex 3D environments for UAV assistive devices for visually impaired individuals. Mnih et al. (2016) proposed the Asynchronous Advantage Actor-Critic algorithm, which adopts multi-threaded asynchronous training without the need for experience replay. Sample efficiency is improved through parallel environmental interaction, providing a fast-training policy gradient method for visually impaired navigation devices. Schulman et al. (2017) proposed Proximal Policy Optimization, which introduces a clipping function to limit the magnitude of policy updates, balancing training efficiency and policy stability. Fast convergence is achieved with limited sample data. Taheri et al. (2024) adopted an enhanced PPO neural network structure, combining residual blocks with a carefully designed reward function. Mobile robots were trained for obstacle avoidance in the Gazebo simulation environment, and control signals were generated by integrating LiDAR data. The success rate was significantly improved, providing a safe navigation solution for visually impaired individuals. Liu et al. (2024) improved the A3C algorithm and combined it with Jump Point Search (JPS) as the global planner. Abstract paths are calculated for global planning, and A3C learns local motion control strategies to coordinate local and global planning. Fujimoto et al. (2018) proposed Twin Delayed Deep Deterministic Policy Gradien, which introduces twin Q-networks, delayed policy updates, and target policy smoothing to solve the overestimation bias problem. The training stability and accuracy are significantly improved. Olayemi et al. (2024) adopted TD3 combined with digital twin perception, eliminating the need for pre-created virtual environments, and ensuring obstacle avoidance and goal-based path planning. Verification was conducted in both simulated and real office environments. Haarnoja et al. (2018) proposed Soft Actor-Critic, which introduces a maximum entropy framework to enhance policy exploration. Entropy regularization is adopted to encourage exploration, achieving excellent performance in highly uncertain environments. Yang et al. (2025) proposed Curriculum Transformer Soft Actor-Critic, which combines curriculum learning to realize goal-directed exploration in complex environments such as underground parking lots. The success rate reaches 0.8, which is 22% higher than that of traditional methods. Kulkarni et al. (2016) proposed Hierarchical DQN, which combines temporal abstraction and intrinsic motivation to realize hierarchical decision-making in robot navigation, providing multi-level path planning capabilities for visually impaired individuals. Levy et al. (2018) adopted curriculum-based DRL, combining hierarchical planning and curriculum learning. A* is used to generate intermediate waypoints, and DRL learns navigation actions. The navigation distance difficulty is gradually increased. Dawood et al. (2025) proposed safe multi-agent reinforcement learning, which combines model predictive control to realize multi-robot collaborative navigation with a zero collision rate and faster goal arrival than baseline methods. Chen W. T. et al. (2025) adopted a multi-agent reinforcement learning planner with centralized training and decentralized execution. A team of quadruped robots collaborates to tow loads through complex environments, which can be scaled up to 12 robots.

### 4.4 Comprehensive comparison of algorithms

To compare the core characteristics of mainstream obstacle avoidance algorithms for obstacle-avoiding robots for the disabled, a multi-dimensional comparison is conducted for the aforementioned five classic algorithms, focusing on the pros and cons of the original algorithms, improvement strategies, and advantages of the improvements, as shown in Table 1.

**TABLE 1:** Comparison table of original and improved obstacle avoidance algorithms.

Algorithm Name	Advantages and disadvantages	Improvement strategy	Improved advantages
Dijkstra’s Algorithm	It is efficient, simple and intuitive, and gradually becomes optimal; however, it has high space requirements and is not suitable for real-time updates	Improve the calculation method; reduce the number of nodes through graphical modeling to optimize the calculation process (Bing and Lai, 2022)	Reduce search nodes and enhance computational efficiency
A* Algorithm	It is optimal and efficient; however, it cannot handle dynamic obstacles and has low search efficiency in complex environments	Optimization of neighborhood expansion (Huang et al., 2025), and removal of redundant points (Xu et al., 2024)	Smoother path and higher search efficiency
RRT Algorithm	The search capability is good, but it is random and inefficient	Gradually optimal (Webb and van den Berg, 2012)	Pathwise gradual optimization and improved smoothness
Dynamic Window Approach (DWA)	High computational efficiency but weak dynamic obstacle prediction	Obstacle movement prediction (Betoño et al., 2025), speed space optimization (Fox et al., 2002)	Strong adaptability to dynamic obstacles and improved real-time performance
Artificial Potential Field (APF)	The computational load is small and the real-time performance is good, but there are defects such as local minima and oscillation	Improved potential field function (sine distance factor) (Zhang et al., 2024)	Solve the local minimum problem, and the path is smoother

### 4.5 Multi-algorithm fusion

A single algorithm is often unable to satisfy multiple requirements simultaneously, such as global optimality, real-time dynamic obstacle avoidance, and human-machine friendliness. Therefore, multi-algorithm fusion has become a mainstream research direction at present. The strategy that combines global and local planning can effectively integrate the advantages of various algorithms. Ma et al. (2025) proposed a hybrid obstacle avoidance algorithm integrating an improved simulated annealing mechanism and an adaptive APF. This improvement significantly enhanced the motion reliability of robots in complex environments. To tackle real-time obstacle avoidance in unstructured assembly environments, Zhang et al. (2025) proposed a two-layer real-time motion planning method combining bidirectional Rapidly-exploring Random Tree (Bi-RRT) and Soft Actor-Critic (SAC). Bi-RRT is used to quickly plan a globally optimal path, while SAC serves as a local obstacle avoidance module to achieve precise dynamic obstacle avoidance. Gao and Li (2025) proposed a hybrid path planning method to address the obstacle avoidance problem of unmanned aerial vehiclesin scenarios with cylindrical obstacles, as illustrated in Figure 12. First, an improved A* algorithm is utilized for global optimal planning. Subsequently, the improved DWA algorithm is invoked during movement to perform real-time obstacle avoidance and local replanning. By means of an adaptively improved evaluation function, this method effectively solves the problems of deadlock and low endurance.

**FIGURE 12:**
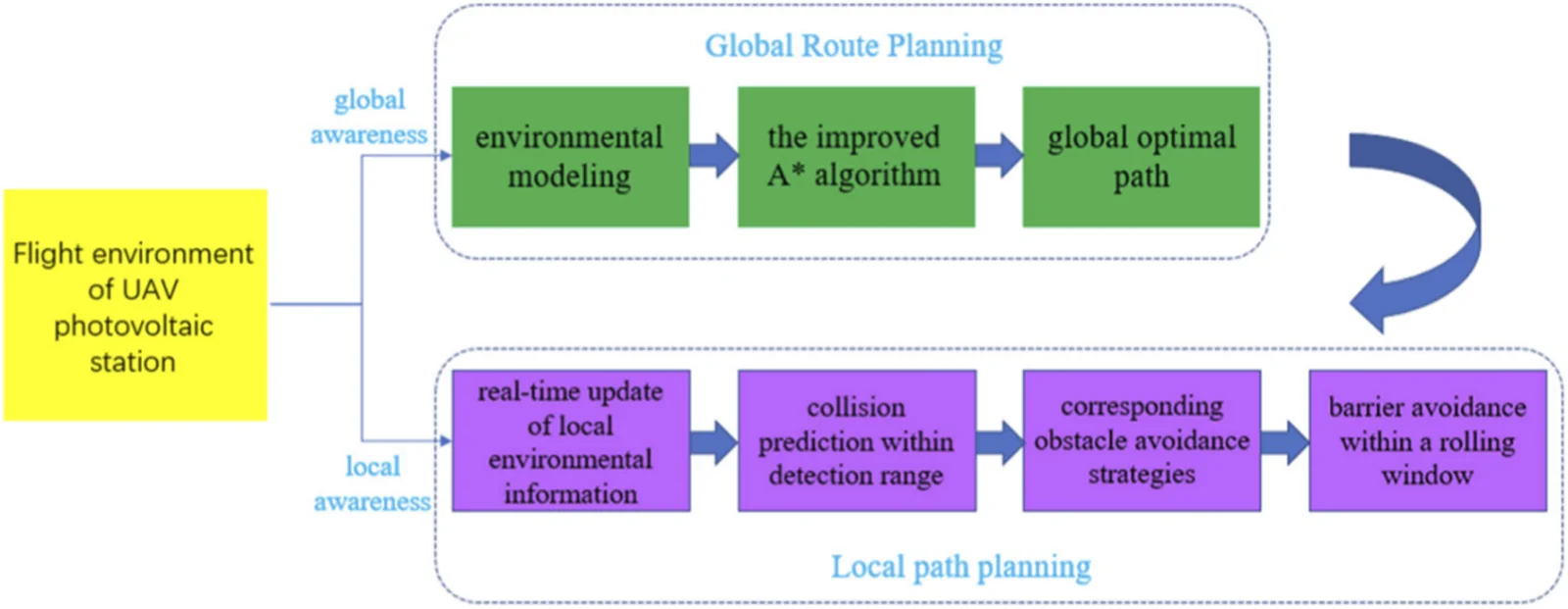
Framework structure diagram of the hybrid path planning method (Gao and Li, 2025).

Yi et al. (2025) proposed an algorithm integrating an enhanced social force model and an improved DWA for Automated Guided Vehicles, and SparkLink communication technology was utilized to improve data transmission speed. This method combined with the social force model enables AGVs to exhibit more flexible performance in the face of dynamic obstacles. Tang Shijun et al. (2025) proposed a noise-suppressed manipulability optimization and obstacle avoidance scheme. A fuzzy adaptive controller was adopted to suppress external disturbances, which not only ensures effective obstacle avoidance, but also prevents the manipulator from falling into singular configurations by optimizing manipulability. Adiuku et al. (2024) leveraged the high precision of YOLOv7 obstacle detection as well as the efficient path planning capability of RRT and DWA to improve the navigation performance of mobile robots in complex and dynamically changing real-world environments, as illustrated in Figure 13.

**FIGURE 13:**
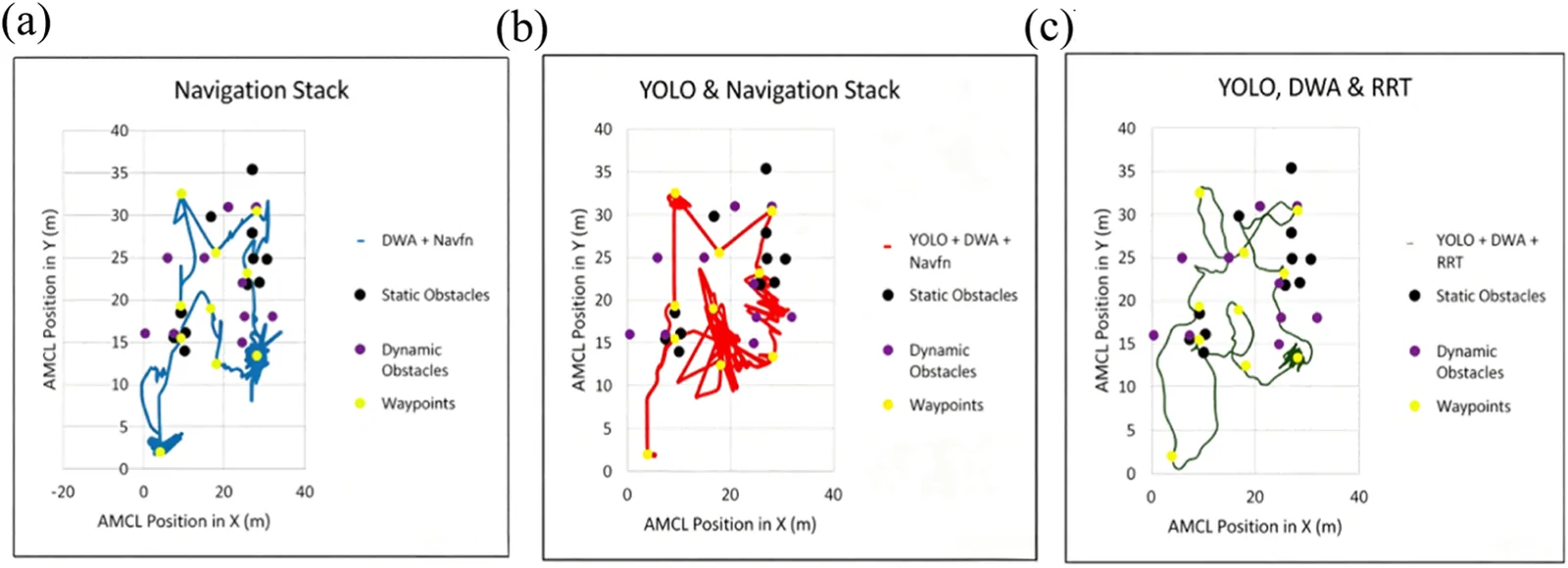
Comparison diagram of trajectories for three navigation schemes (Adiuku et al., 2024). **(a)** ROS Navigation stack trajectory. **(b)** NAV-YOLO trajectory. **(c)** NAV-YOLO-RRT trajectory.

Zheng (2024) designed an autonomous obstacle avoidance and trajectory planning scheme for mobile robots based on double-loop trajectory tracking control and an improved artificial potential field method. The advantages of this method in terms of motion trajectory tracking accuracy and motion stability were verified, demonstrating excellent effectiveness and feasibility. Liu et al. (2021) proposed an improved A*-based path planning algorithm, where artificial potential field factors were introduced into the evaluation function to generate a safe and collision-free initial path. Subsequently, a turning point optimization algorithm based on the greedy algorithm was adopted to reduce the number of path turns. At present, path planning has formed a pattern featuring lightweight upgrading of traditional algorithms and integrated application of intelligent algorithms. Nevertheless, issues such as replanning efficiency in dynamic and complex environments, excessive algorithm conservatism, the depth of personalized path planning, and the smooth connection between planned paths and actual robot movements still need to be further addressed to enhance the practicality of robots.

## 5 Scenario-specific applications

### 5.1 Assistive applications for people with disabilities

The key to implementing assistive technology for the disabled lies in delivering environmental perception information to users in an efficient, comfortable, and perceptually natural manner. Paul et al. (2025) designed an AI-enhanced multifunctional intelligent guiding cane, which integrates ultrasonic sensors, GPS, GSM modules, and AI object detection functions, capable of providing real-time voice alerts and emergency assistance services. Yang et al. (2024) developed an intelligent cane and related sensor system with an ultra-simple structure and extremely low cost based on a triboelectric nanogenerator, which is used for detecting potential hazards and falls. Okolo et al. (2024) developed an intelligent cane based on sensor data fusion and fuzzy logic, which utilizes the Robot Operating Systemarchitecture for real-time processing and guides users through a tactile-voice interface. In the field of wearable devices, Păpară et al. (2024) tested a cost-effective and user-friendly indoor obstacle detection prototype, which can communicate according to user language preferences and alert users to the presence of obstacles through different levels of warnings. The “Expandable Space Glove” developed by Kilian et al. (2022) transmits the relative position and distance of nearby objects to the back of the hand as vibration stimuli, enabling visually impaired individuals to haptically explore the depth of the surrounding space, and it has demonstrated high usability in navigation tasks. Werner et al. (2023) tested four systems including no feedback, auditory feedback, tactile feedback, and multimodal feedback, and found that multimodal auditory-tactile feedback can significantly improve the success rate of visually impaired individuals crossing simulated intersections, with tactile feedback being considered more intuitive by most participants. Through mixed-methods experiments, Kuriakose et al. (2023) found that despite individual preference differences, providing detailed environmental information in audio navigation instructions. Casado and Pedrino (2025) developed a novel monocular depth estimation method based on convolutional neural networks and attention mechanisms, aiming to simplify data collection and processing in navigation assistive systems. Chen et al. (2022) proposed a new quadruped guiding robot system based on the concept of comfort. By designing a controllable traction device to adjust the length and force between humans and robots, comfort is ensured. At present, human-robot interaction has evolved into a multimodal, personalized, and intelligent model, as illustrated in Figure 14. Research on emotion-supportive interaction is also increasing. However, issues such as insufficient practicality of some interaction methods, poor coordination of multimodal interactions, lack of interaction solutions for individuals with severe disabilities, and non-intuitive interaction interfaces still restrict the popularization and application of robots.

**FIGURE 14:**
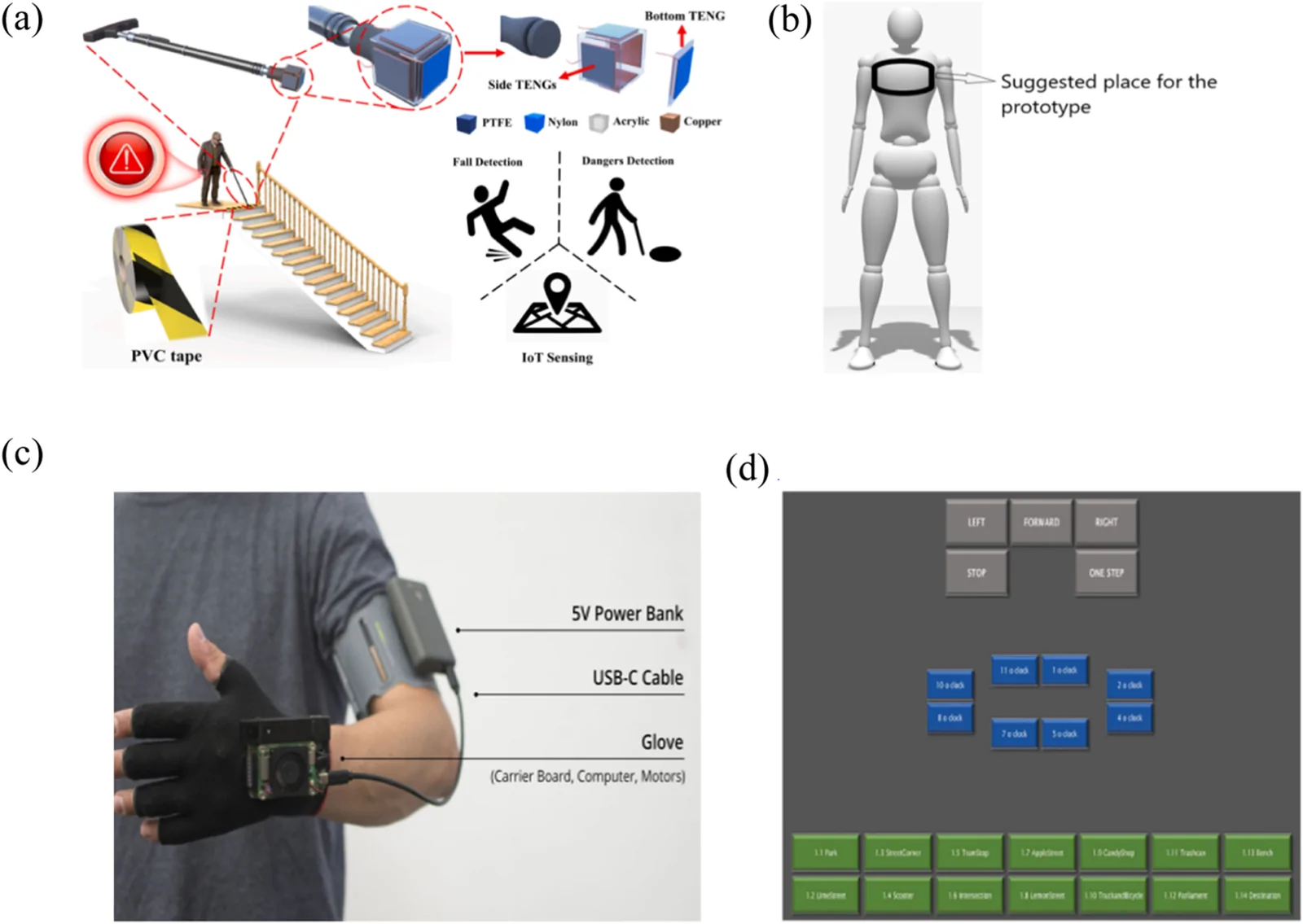
Scenario-based application. **(a)** Schematic of the TENG-based smart cane and its application scenarios (Yang et al., 2024). **(b)** Placed on the torso (Păpară et al., 2024). **(c)** The final prototype with labeled components (Kilian et al., 2022). **(d)** Audio command desktop application (Kuriakose et al., 2023).

### 5.2 Evidence hierarchy and limitations of existing research

Overall, the evidence base remains dominated by early-stage validation. Approximately 10% of the studies reviewed in Sections 2–5 reported real-world deployment with the target user population, 35% reported limited physical testing, 35% reported simulation-only evaluation, and the remaining 20% reported laboratory prototype validation. These proportions are approximate and are intended to provide an overall characterization of the reviewed literature rather than a formal reclassification of every individual study.

There are significant differences in the scientific reliability of evidence in existing literature. Strong evidence comes from long-term real deployment targeting the target user group, such as Ga et al. (2025) conducting a 7 month field deployment on 12 visually impaired volunteers and verifying the results through two factor analysis of variance. However, some studies still remain at a lower level of evidence, relying on pure simulation results. For example, Ahmed and Das (2025) evaluated the performance of YOLOv5 and Faster R-CNN on the KITTI dataset. Although the study reported comparative detection performance, statistical uncertainty measures, such as standard deviations, confidence intervals, or analyses of variance, were not provided for the reported results. Similarly, Tafnakaji et al. (2023) only provided discrete experimental points and calculated averages, but did not record any testing conditions, sample sizes, or statistical methods. Therefore, although these studies provide valuable exploratory insights, their scientific reliability and operational stability for target users have not been confirmed compared to real-world field trials.

## 6 Conclusion

Through the review and analysis of existing relevant literature, the field of assistive robots for the disabled has gradually formed a research framework encompassing various key technologies after years of exploration and development. This framework primarily includes environmental perception, obstacle recognition and classification, path planning and obstacle avoidance algorithms, as well as scenario-specific applications. Existing literature indicates that the field is transitioning from a single-function prototype verification stage to a new stage of systematization, intelligence, and scenario adaptability.

In terms of environmental perception, the research paradigm has shifted from single sensor to multi-sensor fusion (LiDAR, depth camera, ultrasonic sensor), significantly enhancing the accuracy of 3D mapping and robustness of localization in complex environments, achieving precise localization and real-time reconstruction in both indoor and outdoor scenarios. With the introduction of deep learning and semantic segmentation technology, obstacle recognition has evolved from simple distance measurement to semantically rich intelligent perception, enabling efficient obstacle classification, motion state prediction, and fine recognition of static and dynamic targets. In path planning and obstacle avoidance, some studies attempt to combine mature global planning and local obstacle avoidance techniques with reinforcement learning and multi-algorithm fusion strategies to ensure the safety and optimality of paths, flexibly adapting to complex travel scenarios for the disabled. Applications for specific scenarios have diversified, including intelligent guide robots for the blind, intelligent wheelchairs, and indoor service robots. Related research covers different scenarios such as homes, hospitals, and urban roads, but its application scope and maturity vary depending on the scenario.

In summary, assistive robots for the disabled possess significant potential for optimization in terms of environmental understanding and task planning capabilities. The iterative upgrading of related technologies has also propelled the evolution of these devices from functional assistive tools to intelligent companions and assistive devices, providing technical support for the construction of a barrier-free and inclusive society.
